# Mitochondrial Gene Expression of Three Different Dragonflies Under the Stress of Chlorpyrifos

**DOI:** 10.3390/insects16010085

**Published:** 2025-01-16

**Authors:** Yuxin Chen, Ziwen Yang, Zhiqiang Guo, Lemei Zhan, Kenneth B. Storey, Danna Yu, Jiayong Zhang

**Affiliations:** 1 College of Life Sciences, Zhejiang Normal University, Jinhua 321004, China; 2 Department of Biology, Carleton University, Ottawa, ON K1S 5B6, Canada; 3 Key Lab of Wildlife Biotechnology, Conservation and Utilization of Zhejiang Province, Zhejiang Normal University, Jinhua 321004, China

**Keywords:** Chlorpyrifos, mitochondrial gene transcript level, dragonfly

## Abstract

Chlorpyrifos (CPF) is an organophosphate insecticide that is extensively utilized globally due to its effectiveness against over 200 pest species. CPF exhibits its toxicity primarily through the inhibition of the acetylcholinesterase (AChE) enzyme, while mitochondrial damage and dysfunction have also been observed. The present study quantified the transcript levels of mitochondria protein-coding genes (mtPCGs) using quantitative real-time polymerase chain reaction (*RT-qPCR*) in samples of larvae of three dragonfly species (*A. parthenope*, *E. elegans*, and *G. confluens*) under different levels of CPF stress. By exposing larvae from uncontaminated populations to 0.05 μg/L CPF for 24 h, the transcript levels of seven mtPCGs in *A. parthenope* were significantly increased (*p* < 0.05) by 1.89 ± 0.42-fold for *COI*, 4.30 ± 0.24-fold for *COIII*, 5.94 ± 0.17-fold for *ND1*, 4.69 ± 0.56-fold for *ND2*, 3.44 ± 0.48-fold for *ND4*, 2.19 ± 0.53-fold for *ND4L*, and 5.05 ± 0.36-fold for *Cytb*, respectively. In *E. elegans*, the transcript levels of *ND1*, *ND2*, and *ND4* increased by 1.23 ± 0.15, 1.48 ± 0.31, and 1.98 ± 0.25-fold, respectively (*p* < 0.05). In *G. confluens*, the transcript levels of *COI*, *COIII*, and *ND4* increased by 1.56 ± 0.13, 1.50 ± 0.26, and 3.74 ± 0.40-fold, respectively (*p* < 0.01). It was demonstrated that the transcript levels of different mtPCGs showed significant up-regulation in the three different dragonfly larvae under CPF stress in the absence of mortality. *ND4* was significantly increased in all three species, indicating that it is an important target gene. The present study underscores the response of mitochondrial gene expression in larvae of three different species in response to CPF pollutants, indicating that pesticide influences can potentially alter mitochondrial gene expression and potentially act as a method for assessing aquatic ecosystem health.

## Simple Summary

Mitochondrial genes can play a crucial role in insect resistance to pesticides. This study investigated the impact of chlorpyrifos (CPF) insecticide on the mitochondrial function of dragonflies by measuring changes in the transcript levels of mitochondrial protein-coding genes (mtPCGs) using the larvae of three dragonfly species (*Anax parthenope*, *Epophthalmia elegans*, and *Gomphidia confluens*). The study demonstrated that transcript levels of different mtPCGs from the three dragonfly species were significantly elevated in the presence of CPF, reflecting that these genes respond to varying levels of environmental contamination. These findings provide a new direction for pesticide detection in aquatic environments.

## 1. Introduction

Chlorpyrifos is utilized in agricultural pest control through mechanisms of contact, ingestion, and gut toxicity, and fumigation [1]. Its toxicity is associated with various physiological and lifestyle-related disorders. These include neurological issues, endocrine disruption, hematological abnormalities, and reproductive problems [2]. CPF blocks the acetylcholinesterase (AChE) active site and interacts with neurotransmitter receptors. This leads to adverse neurological effects and significant toxicity in non-target organisms [3,4]. CPF primarily exerts its toxic effects by inhibiting AChE enzymes. These enzymes are involved in modulating neurotransmission through the hydrolysis of acetylcholine (ACh) [1,5]. Inhibition leads to the accumulation of Ach and over-stimulation of Ach receptors, potentially causing altered behavioral states, paralysis, and even death in exposed insects [6,7]. It has been shown that CPF stress alters the behavioral state of insects [8,9,10]. Despite its efficacy in pest control and subsequent improvement in crop yields, CPF has posed significant health risks to humans and other terrestrial animals. Additionally, unreasonable use of this chemical has led to environmental problems. The extensive application of CPF can lead to water contamination, with its presence detected in various aquatic environments such as rivers, lakes, seawater, and even rainfall [11].

One of the primary mechanisms underlying pesticide toxicity has been proposed to involve mitochondrial dysfunction [12]. CPF can induce mitochondrial dysfunction through several mechanisms, which can lead to a variety of adverse effects. The primary mechanism involved is the inhibition of AChE, but CPF also affects mitochondria directly. It can cause oxidative stress, which is one of the key factors leading to mitochondrial damage [13,14]. This stress is triggered by the generation of reactive oxygen species (ROS) that overwhelm the antioxidant defenses of cells, leading to lipid peroxidation, protein oxidation, and damage to mitochondrial genes [15,16,17,18]. To evaluate the long-term risks associated with CPF exposure more effectively, studies of its impact on mitogenomes are crucial.

Mitochondria are essential organelles within eukaryotic living organisms and are the primary sites responsible for both oxygen consumption and adenosine triphosphate (ATP) production, which serves as the energy currency of cells. Mitogenomes can facilitate phylogenetic, comparative genomic, and genetic barcoding analyses. As more and more mitogenomes have been sequenced (providing important sequence information), data have also been gathered on other aspects of mitochondrial genes, including replication strategies [19,20], transcriptional and translational architectures [21,22], mutational information [23,24,25], and arrangements [26,27,28,29,30]. Mitochondria adjust their oxidative phosphorylation process and regulate ATP production rates in response to environmental fluctuations, ensuring the maintenance of biophysiological stability [31,32]. CPF-induced apoptosis is associated with mitochondrial dysfunction through the generation of ROS [33,34]. ROS can impair mtDNA [35], membrane lipids [36], and proteins [37]. Additionally, environmental stressors can exacerbate these effects. Temperature fluctuations and chemical exposures generate unique DNA adducts, leading to significant mitochondrial dysfunction [38,39,40,41]. At extreme temperatures, mitochondrial activity adjusts to minimize damage, manifesting as alterations in gene expression, mtDNA levels, or phosphorylation efficiency [41,42,43,44,45,46]. Similarly, *COI* and *12S rRNA* transcript levels decreased in the larvae of goldenrod gall insects under fluctuating oxygen levels [47]. Guan et al. [48] reported that exposure to imidacloprid resulted in altered mitochondrial gene expression in *Choroterpes yixingensis*. This finding underscores the vulnerability of aquatic insects, particularly when environmental contaminants such as pesticides are flushed into aquatic environments, disrupting mitochondrial energy metabolism and causing mtDNA damage [1,49,50,51,52]. Several studies have reported that CPF disrupts the electron transport chain (ETC) by impairing complex I and reducing antioxidant defenses essential for scavenging free radicals, leading to an increase in mitochondrial gene expression [34,53,54,55]. While extensive research has been conducted on stress resistance mechanisms in insects, much of the focus remains on detoxification processes and nuclear genome target genes. The response of the mitochondrial gene to insecticide stress remains an area that requires further investigation.

As hemimetabolous insects, dragonflies undergo a lifecycle that transitions from aquatic larvae to terrestrial adults, making them uniquely positioned to assess both aquatic and terrestrial environments. Although adults inhabit terrestrial environments, the larvae remain highly dependent on aquatic habitats and are extremely sensitive to changes in environmental conditions [56]. Due to these characteristics, dragonflies often serve as indicator species for evaluating ecological conditions [57]. Their observable biological responses enable intuitive assessments of local water quality and pollution levels [57]. Chang et al. found that CPF can disrupt physiological processes, potentially leading to long-term impacts on fitness and survival rates of species such as *Ceriagrion* species when exposed to environmental chemical stressors [58]. Furthermore, by assessing the species richness, abundance, and diversity of dragonflies, researchers have established a negative correlation between water pollution levels and dragonfly diversity [59,60]. Water contamination has also been quantified by analyzing the accumulation of heavy metals in dragonfly larvae [61]. Consequently, Odonata has been used as a standard environmental assessment tool for wetlands, as outlined in the Integrated Wetland Assessment Toolkit published by the International Union for Conservation of Nature (IUCN) in 2009 [62]. However, due to the varying environmental preferences and biological habits of the aquatic larvae of different dragonfly species, tolerance levels to environmental chemicals differ. Consequently, establishing an appropriate assessment standard presents a significant challenge. Species such as *Ischnura elegans* (Odonata: Zygoptera), which thrive in standing waters, exhibit tolerance to pollution and eutrophication [63]. This adaptability allows them to allocate part of their energy towards repairing and maintaining physiological integrity, highlighting the variability in species responses to environmental chemicals [64]. Even species that are closely related can exhibit markedly different levels of sensitivity to a pesticide [65]. Thus, the present study focuses on investigating the mitochondrial gene expression of three dragonfly species (*Anax parthenope*, *Epophthalmia elegans*, and *Gomphidia confluens*) under different concentrations of CPF stress using *RT-qPCR*. This work can enhance our understanding of the role that mitogenomes play in organismal tolerance to polluted environments.

## 2. Materials and Methods

### 2.1. Specimen Collection and CPF Treatment

Dragonfly larvae were all collected in the Agula Wetland Park from Tongliao (N 121°59′, E 42°59′), Inner Mongolia autonomous region, China. Specimens were identified at the species level based on the morphological features of the anterior, antenna, and anal appendages [66]. To minimize physiological and metabolic variations among larvae of different stages, we used a digital caliper to measure the head width and total length of each specimen (Table 1), ensuring that they were all in the ultimate instar. We identified that all specimens could be divided into *A. parthenope*, *E. elegans*, and *G. confluens*. After species identification, larvae were transferred to aerated aquaria at a controlled temperature of 24 °C (natural habitat) for one week to acclimate before exposure to experimentation. Larvae were fed by Hulk orange shrimp (*Neocaridina denticulata*) every day.

We first investigated the effect of low CPF concentration (0.05 µg/L), which is below the allowable environmental limit set in the United States [69], on the mitochondrial gene transcript levels of the three dragonfly species. We divided the 40 dragonfly larvae of each species into a control group and the three experimental groups (0.05 μg/L, 0.5 μg/L, and 5 μg/L), with ten larvae per group. Each group with ten larvae was bred in aquaria (80 × 50 × 50 cm) filled with 100 L of the aerated water. Moreover, CPF pesticide (CAS-No.: 2921-88-2, purity 98%; Shanghai Canspec Scientific Instruments Co., Ltd., Shanghai, China) was diluted with uncontaminated water and ethanol (analytical purity) to 500 mL at 5 μg/mL. The final test concentrations (0.05 μg/L, 0.5 μg/L, and 5 μg/L) were obtained by serial dilutions from a stock solution of 5 g/mL (uncontaminated water + ethanol). Based on the environmental allowable limit in the United States [69], the experimental groups were set to culture for 24 h under 0.05 μg/L, 0.5 μg/L, and 5 μg/L CPF, and the controls were kept in the same solution but CPF-free. We randomly selected four surviving individuals from each group (the individuals with similar genetic sequences) to be placed in an RNase-free environment rapidly dissected to obtain 15 ± 0.1 mg of gut tissue from individual specimens, which were immediately frozen in liquid nitrogen. Then, they were stored at −80 °C in the laboratory of JY Zhang, College of Life Sciences, Zhejiang Normal University, for further experiments.

To verify that the target genes obtained from our screening at low concentrations (0.5 μg/L) of CPF were effective target genes of CPF, we further tested these target gene expressions from larvae subjected to the 0.5 μg/L and 5 μg/L CPF stress, respectively. With other experimental conditions unchanged, the concentration of CPF stress was varied to determine if significant up-regulation of the target genes was also observed in the 0.5 μg/L and 5 μg/L CPF experimental groups.

### 2.2. DNA Extraction and Sequencing

Selection of all samples from each group of the three species for DNA extraction was performed. Muscle tissue from the legs of specimens was processed using the Ezup Column Animal Genomic DNA Purification Kit (Sangon Biotech Company, Shanghai, China). DNA barcoding was amplified using universal *COI* primers as described in Black et al. [70] with the forward primer (5′-GGTCAACAAATCATAAAGATATTGG-3′) and reverse primer (5′-TAAACTTCAGGGTGACCAAAAAATCA-3′). In addition, *β-actin* from the three species was chosen as the reference gene and successfully amplified by using forward primer (5′-CTTCCTTCCTGGGTATGG-3′) and reverse primer (5′-GCGGTGATTTCCTTCTGC-3′) [48]. Successful amplification of the target genes of the three species was evaluated using agarose gel electrophoresis. Then, all PCR products were sequenced by Youkang Biotech (Hangzhou, China). SeqMan in the DNASTAR package v.7.1 [71] was used to manually check and assemble Sanger sequencing results. The DNA samples of one individual of *A. parthenope*, *E. elegans*, and *G. confluens* were sequenced to obtain their mitogenomes using next-generation sequencing (NGS) at BGI Tech Inc. (Shenzhen, China). The obtained *COI* sequences served as a reliable indicator that the extracted DNA sample was free from contamination.

### 2.3. Mitogenome Assembly and Annotation

The complete mitogenomes of the three species were manually assembled using MitoZ [72], NOVOPlasty v.4.2 [73], and GetOrganelle v.1.7.1 [74], respectively, to ensure the accuracy of the results. The MITOS service (http://mitos.bioinf.uni-leipzig.de/index.py, accessed on 20 October 2023) [75] was used to determine the gene regions of tRNA and predict tRNA secondary structures. The sequence alignments with homologous genes of *A. parthenope* MT371045, *E. elegans* MK522522, and *Davidius lunatus* EU591677 were assessed using ClustalW in Mega v.7.0 [76] and could not only identify rRNA genes (*12S rRNA* and *16S rRNA*) but also approximate the length and regions of the 13 mtPCGs. Subsequently, we manually annotated the approximate location of the 13 mtPCGs. The mitogenomes of *G. confluens*, *A. parthenope*, and *E. elegans* were deposited in the NCBI, with accession numbers PP577161, PP577162, and PP577163, respectively.

### 2.4. RNA Extraction, cDNA Synthesis, Primer Design

Following the instructions provided in the TaKaRa Mini BEST Universal RNA Extraction Kit (TaKaRa, Tokyo, Japan), we used four individuals per experimental group and the control group of three species with similar genetic sequences to extract RNA from the whole gut. The total RNA concentration was determined using an infinite M200pro enzyme label (Tecan, Ltd., Männedorf, Switzerland), and RNA purity was appraised using the A260/280 ratio (1.8~2.1). Then, we stored the RNA in an ultra-low temperature refrigerator at −80 °C.

**Table 2:** Primer sequences of the 10 mitochondrial PCGs used for *RT-qPCR* in this study. The β-actin gene was used as the reference gene.

Gene	Species	Gene Forward Primers (5′ to 3′)	Reverse Primers (5′ to 3′)
*β-actin*	*A. parthenope*	GAAACCGTCTACAACTCAATCA	GCATCCTGTCAGCAATACC
	*G. confluens*	AACCGTCTACAACTCAATCA	TCCTTCTGCATCCTGTCA
	*E. elegans*	ACCGTCTACAACTCCATCAT	GCATCCTGTCAGCAATACC
*COI*	*A. parthenope*	TAGGAGCACCCGATATAGC	CACCAGCAAGAGGAGGAT
	*G. confluens*	ATACCACGACGATACTCTGA	GTGCTGCTATGGCTTCTC
	*E. elegans*	GCTGGAATGGTTGGAACA	CGTGTGCTGTGACAATAAC
*COII*	*A. parthenope*	GCTTGAACTGTCCTTCCA	ACCACTGATGTCCTACTGT
	*G. confluens*	GAAGTAGATAACCGAGCAGTT	GTCCTGGAGTTGCGTCTA
	*E. elegans*	AGGAGGACTTCGCTTACTT	GTGTAGCATCAACCTTAACTC
*COIII*	*A. parthenope*	TTCACAGAAGTCTATCTCCAAC	GTTACAGTTACTCCTGATGCTA
	*G. confluens*	TGGTAATTGGACTACGATGAG	CCTTAGGAGGTCATATACTTCC
	*E. elegans*	ACCATTCACAATCGCAGAT	AAGGCAATGTCGTATTAAGC
*ND1*	*A. parthenope*	GGTGGAGGATATATACTTGATG	GCTAAACAAGACGCAAATCA
	*G. confluens*	TTGCTGGTTGGGCTTCTAA	CTCGTAAGCCTCCCAATAAAG
	*E. elegans*	TGCTGGATGAGCTTCTAATTC	TATGAAATAGTCTGAGCCACAG
*ND2*	*A. parthenope*	GCTGTAGGAGGACTTAATCA	CCAAGATGTCTAATGGAAGA
	*G. confluens*	CCAAGCTATTGCTTCAGTAATC	GGAAATCAGAAATGGAAAGGAG
	*E. elegans*	ATTTCCGGGAGTAATAGAAGG	GTGTAGATAGAAGGACTGAAGT
*ND3*	*A. parthenope*	CCACTGCACGTATTCCTT	GCACCTTGATTTCATTCGT
	*G. confluens*	CCTACGATTCTTCTTGATTGC	GGTAATGTTTGATGCGGTAAG
	*E. elegans*	GCACGAATCCCATTCTCTTTA	AAGTAGGGCAATTTCCACATC
*ND4*	*A. parthenope*	GGATTAAGTGGTGCCTATACT	ATAAACTACGACTCCCAAGAC
	*G. confluens*	GCTCCTATTTCTGGATCTATGA	CAACAACTCCTCCTACTAATCT
	*E. elegans*	GAGTTGGTATTGCTTTATTGGG	ACCTATATGAGCCACAGAAGA
*ND5*	*A. parthenope*	GATAGAGTTGAAGCCCAAGTTA	AAGGCAGATACAGGAGTAGG
	*G. confluens*	TTAGGATGAGATGGGTTAGGT	CGATTAGAAAGCACAGTCAAC
	*E. elegans*	ATGACTAAGAGTGCTCAGATTC	AAGCAGATACAGGAGTAGGAG
*Atp6*	*A. parthenope*	GGAACTACAGGACATAATGGAA	GTGAATGCTAGATGACTTGAAC
	*G. confluens*	GCAGGACATCTTCTAATAACTC	GCTACTGCGGATTCTAATACT
	*E. elegans*	TTACCAGAACCAGTCACATATC	CTGAGGAACTAAATGAGCAAAC
*Cyt b*	*A. parthenope*	GGGTGATTATTACGAACTCTTC	CTACTCCTACACTTCAAGTATG
	*G. confluens*	CGGATGACTTCTACGAACAT	CTCCAACTCTTCAAGTGTGA
	*E. elegans*	CTGAGGAGCCACCGTAAT	GGCGTTATCTACTGCGAAT

According to the manufacturer’s instructions, 2 μL of 5 × gDNA Eraser Buffer, 1 μL gDNA Eraser, and 7 μL of a mixture containing RNase-Free ddH_2_O and RNA were mixed to a final volume of 10 μL and then heated at 42 °C for 2 min. Each mixture was then mixed with 1 μL Primer Script RT Enzyme Mix I, 1 μL RT Primer Mix I, 4 μL 5 × PrimerScript Buffer 2, and 4 μL RNase-free ddH_2_O to be reverse transcribed into cDNA. The reaction procedure was conducted at 37 °C for 15 min (reverse transcription reaction), 85 °C for 5 s (reverse transcriptase inactivation), and then stored in an ultra-low temperature refrigerator at −80 °C.

Primer Premier v.6.0 software (Premier Biosoft International, Palo Alto, CA, USA) was used to design valid primers for *RT-qPCR* based on the sequences of the 13 mtPCGs and the internal reference *β*-*actin*. The *ATP8* fragment was too short for effective primer design, and *ND3* and *ND6* primers exhibited insufficient amplification efficiency for further experimental use. As a result, ten pairs of primers for the remaining PCGs as well as the *β*-*actin* gene are shown in Table 2, and all primers were synthesized by Sangon Biotech (Shanghai, China).

The reaction mixture configuration followed the method described by Guan et al. [48]: 2 μL cDNA, 6 μL ddH_2_O, 10 μL SYBR Premix Ex Taq II (2×), 0.4 μL ROX Reference Dye (50×), and 0.8 μL of each forward and reverse primer. The *RT-qPCR* conditions were 95 °C for 30 s, followed by 40 cycles of 95 °C for 5 s and 52 °C for 30 s, with melting curve analysis performed at 95 °C for 15 s, 60 °C for 1 min, and 95 °C for 15 s. In particular, samples for primer screening were diluted to five gradient concentrations, run in three technical replicates, and analyzed on an Applied Biosystems 7500 instrument. The results were assessed using StepOne software v.2.2.2 (Applied Biosystems, Foster City, CA, USA), and 11 pairs of fluorescent quantitative primers, including the *β*-*actin* gene, were validated based on the standard and melting curves (melting curves showing a single peak, standard curve *R*^2^ > 0.980, and *E* value between 90 and 110).

### 2.5. The Relative Transcription Levels of mtPCGs

The cDNAs obtained from RNA extraction and reverse transcription were diluted 10-fold. Subsequently, transcription levels were measured for 10 mtPCGs, with each gene repeated three times for four individuals from the experimental and control groups of each species. StepOne software v.2.2.2 (Applied Biological Systems, Foster City, CA, USA) was used to view and export the CT mean value. Gene expression was calculated using the 2−∆∆Ct method with *β-actin* as the reference gene. SPSS v.22.0 (SPSS, Inc., Chicago, IL, USA) was used to calculate. Values for each group are expressed as mean ± SE. Statistical differences between the CPF-treated samples at 0.05 μg/L, 0.5 μg/L, and 5 μg/L concentrations and the control group (without CPF) were evaluated using an independent *t*-test, where *p* < 0.05 was considered a significant difference. Finally, Origin v.8.0 software (Origin Lab, Northampton, MA, USA) and the pheatmap package in *R* software v.4.4.2 were used for data analysis and charting.

## 3. Results

### 3.1. Mitochondrial Gene Expression Under 0.05 µg/L CPF Stress

**Figure 1:**
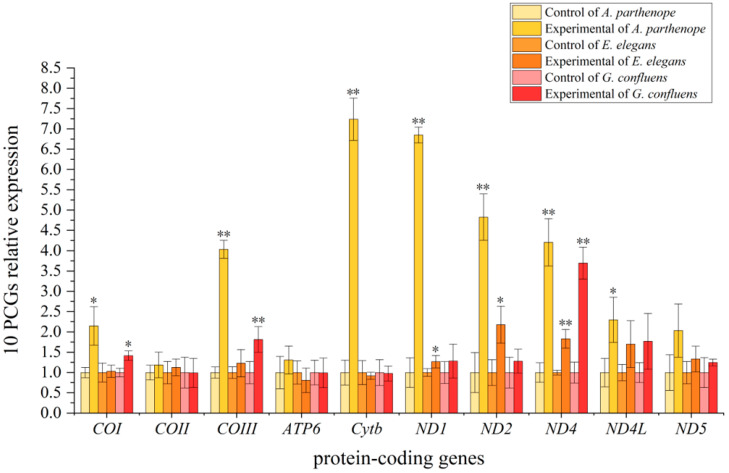
The expression of 10 PCGs from larvae of *A. parthenope*, *G. confluens*, and *E. elegans* comparing control versus experimental conditions (under 0.05 μg/L CPF stress). The x-axis shows gene names. For each gene, 3 pairs of bars are shown. The first (yellow), third (orange), and fifth (red) bars show controls (all standardized to 1.0), whereas the second (yellow), fourth (orange), and sixth (red) bars show the relative gene expression of the experimental data compared to control values. Yellow columns show data for *A. parthenope*; orange columns show data for *E. elegans*; and red columns show data for *G. confluens*. The error bars represent standard deviation. Asterisks indicate significantly different expressions: *, *p <* 0.05; **, *p <* 0.01. The value of *p* was calculated by an independent *t*-test.

For *A. parthenope*, the transcript levels of *COI*, *COIII*, *ND1*, *ND2*, *ND4*, *ND4L*, and *Cytb* were significantly up-regulated (*p <* 0.01). Among them, the magnitude of the upward trend was high, increasing by 4.31 ± 0.24, 5.94 ± 0.17, 4.69 ± 0.56, 3.44 ± 0.48, and 5.05 ± 0.36 fold for *COIII*, *ND1*, *ND2*, *ND4*, and *Cytb*, respectively. The transcript levels of *COI* and *ND4L* genes were also significantly increased by 1.89 ± 0.42 and 2.19 ± 0.53 fold. In *E. elegans*, *ND1*, *ND2*, and *ND4* were significantly increased by 1.23 ± 0.15, 1.48 ± 0.31, and 1.98 ± 0.25 fold (*p <* 0.05), respectively. For *G. confluens*, *COI*, *COIII*, and *ND4* were significantly increased by 1.56 ± 0.13, 1.50 ± 0.26, and 3.74 ± 0.40 fold (*p <* 0.05), respectively (Figure 1).

### 3.2. Test the Target Gene Expressions Under 0.5 µg/L and 5 µg/L CPF Stress

**Figure 2:**
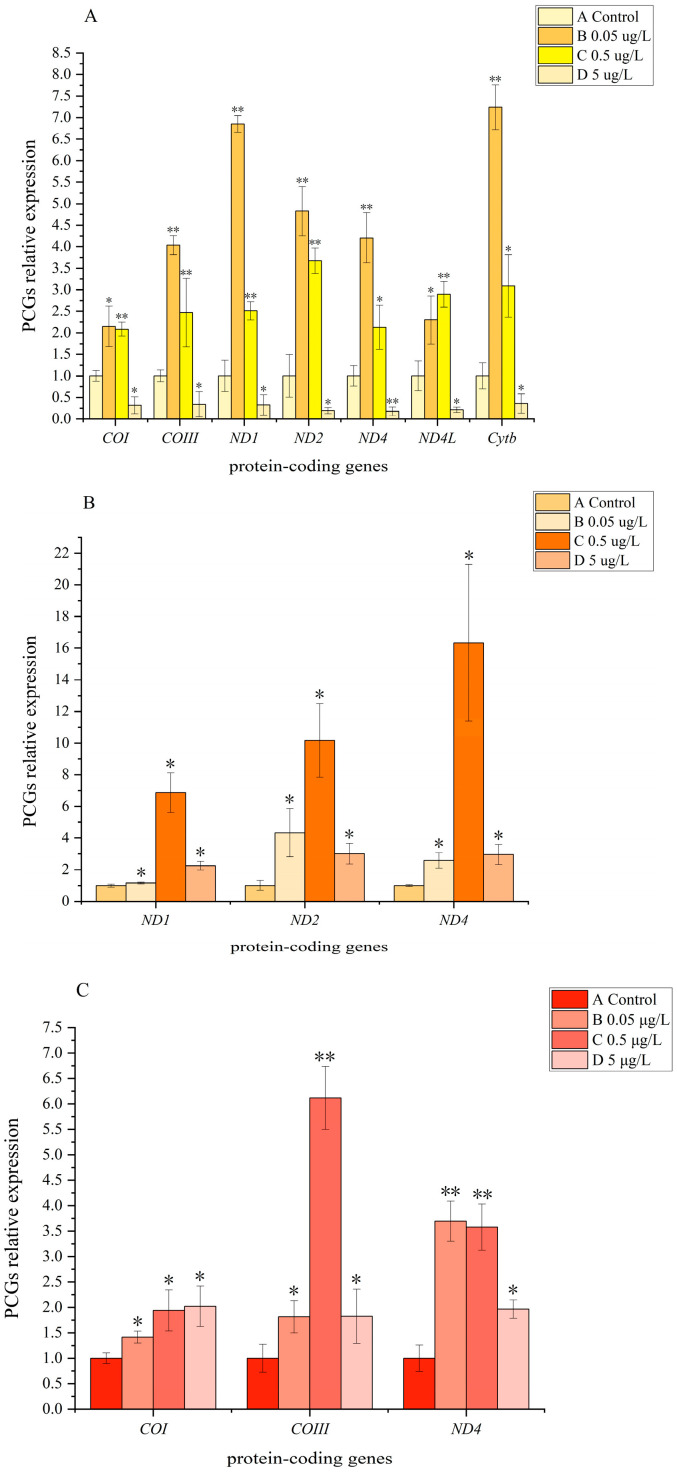
The expression of 10 PCGs from larvae of (**A**) *A. parthenope*, (**B**) *G. confluens*, and (**C**) *E. elegans* comparing control versus experimental conditions (under 0.05 μg/L, 0.5 μg/L, and 5 μg/L CPF stress). The x-axis shows gene names. For each gene, four bars show the relative gene expression of the four experimental conditions compared to control values. The error bars represent standard deviation. Asterisks indicate significantly different expressions: *, *p* < 0.05; **, *p* < 0.01. The value of *p* was calculated by an independent *t*-test.

To further investigate the expression mechanisms of genes with up-regulated transcript levels under 0.05 μg/L CPF stress, we further assessed the transcript levels of up-regulated genes under 0.5 μg/L and 5 μg/L CPF stresses. After 24 h all larvae of *G. confluens* and *E. elegans* were alive, whereas one larva of *A. parthenope* occurred death under 5 μg/L chlorpyrifos stress. In the 0.5 μg/L CPF group of *A. parthenope*, the transcript levels of several genes were still up-regulated significantly (*p <* 0.05). The transcript levels of *COI*, *COIII*, *ND1*, *ND2*, *ND4*, *ND4L*, and *Cytb* significantly increased by 1.83 ± 0.14, 2.63 ± 0.84, 2.18 ± 0.19, 3.57 ± 0.28, 1.74 ± 0.42, 2.75 ± 0.28, and 2.15 ± 0.51 fold, respectively (Figure 2). However, in the 5 μg/L experimental group, the transcript levels of *COI*, *COIII*, *ND1*, *ND2*, *ND4*, *ND4L*, and *Cytb* showed significant levels of reduction (*p* < 0.01), with the transcript levels of 0.28 ± 0.17, 0.36 ± 0.31, 0.28 ± 0.20, 0.19 ± 0.07, 0.15 ± 0.08, 0.20 ± 0.06, and 0.25 ± 0.16 fold, respectively (Figure 2). For *E. elegans*, the genes of *ND1*, *ND2*, and *ND4* were also significantly up-regulated (*p <* 0.05). The transcript levels of *ND1*, *ND2*, and *ND4* significantly increased by 6.68 ± 1.21, 6.89 ± 1.57, and 17.67 ± 5.35 fold, respectively (Figure 2). In the 5 μg/L experimental group, the transcript levels of *ND1*, *ND2*, and *ND4* significantly increased by 2.19 ± 0.27, 2.04 ± 0.44, and 3.19 ± 0.69 fold, respectively (Figure 2). For *G. confluens*, both the experimental groups treated with 0.5 μg/L and 5 μg/L CPF were found to be significantly up-regulated in *COI*, *COIII*, and *ND4* (*p <* 0.05) (Figure 2). In the 0.5 μg/L experimental group, the transcript levels of *COI*, *COIII*, and *ND4* were significantly increased by 2.14 ± 0.45, 5.06 ± 0.51, and 3.62 ± 0.46 fold, respectively (Figure 2). In the 5 μg/L experimental group, the transcript levels of *COI*, *COIII*, and *ND4* were increased by 2.23 ± 0.44, 1.51 ± 0.44, and 1.99 ± 0.18 fold, respectively (Figure 2).

## 4. Discussion

In this study, we initially investigated the transcript levels of 10 PCGs in three species of dragonfly larvae (*A. parthenope*, *E. elegans*, and *G. confluens*) when exposed to 0.05 μg/L CPF stress, a value that is under the environmental allowable limit in the United States [69]. The results indicated that CPF stress predominantly affected complex IV (*COI* and *COIII*), complex I (*ND1*, *ND2*, *ND4*, and *ND4L*), and complex III (*Cytb*) in *A. parthenope*. For *E. elegans*, CPF stress primarily affected complex I, as indicated by the elevated transcript levels of *ND1*, *ND2*, and *ND4*. For *G. confluens*, the higher transcript levels of *COI*, *COIII*, and *ND4* suggested that CPF stress mainly impacted complex I and complex II. As the largest and first complex in the ETC, Complex I is crucial for oxidative phosphorylation and the transfer of electrons from NADH to coenzyme Q10 (CoQ10), which is essential for respiratory metabolism in many aerobic organisms [77,78]. Complexes III and IV collaborate with Complex I in this electron transfer [79], while CPF and other OPs can impair mitochondrial function by disrupting the ETC and oxidative phosphorylation [13,14]. When cells are exposed to CPF, the mitochondria become targets with ROS levels increasing significantly [80,81,82]. The increased expression of mitochondrial genes may be a response aimed at restoring mitochondrial function and mitigating damage by enhancing the repair mechanisms or by upregulating proteins involved in the ETC to compensate for reduced energy production [83].

In particular, we found that the *ND4* gene exhibited significant upregulation in transcript levels in all three dragonfly species under CPF stress. This aligns with findings from Wang et al. [84], who reported elevated *ND4* expression in porcine granulosa cells exposed to malathion, another OP, suggesting that *ND4* upregulation may be a general response to OP-induced mitochondrial stress. Such mitochondrial stress responses are frequently observed in studies addressing both endogenous and exogenous antioxidant damage. For instance, Yan et al. [85] demonstrated that upregulation of mitochondrial *ND4* expression alleviated exogenous oxidative damage in rats. The stress response, essential for preserving cellular homeostasis under oxidative stress, is predominantly regulated by heat shock proteins (Hsps) [86,87]. These proteins are typically upregulated under CPF-induced stress, a phenomenon observed in both fish and insects [1,88]. According to Cui et al., increased expression of mitochondrial chaperones such as *Hsp75* serves as a compensatory mechanism to address mitochondrial dysfunction, either by assisting in protein refolding or promoting the degradation of damaged proteins [18]. Therefore, we believe that the upregulation of *ND4* may be an effective cellular strategy to reduce ROS production and restore mitochondrial function. This suggests their potential involvement as target genes for monitoring exposure to insecticides.

Under higher concentrations (0.5 μg/L and 5 μg/L) of CPF, the target gene expressions were significantly induced. This suggested that these genes can indicate CPF contamination in a range of concentrations in the environment. However, among the three dragonfly species, only *A. parthenope* displayed a notable reduction in the transcript levels of genes and experienced larval mortality under 5 μg/L CPF stress (with one larva dying out of ten individuals). The sharp decline of genes in transcription levels and the mortality of larvae may be due to mitochondrial dysfunction induced by high CPF concentrations or through neurotransmission deterioration and oxidative damage [13]. Multiple studies found that increasing CPF exposure time or concentration leads to a corresponding rise in mortality, a decline in the activity of energy metabolism enzymes, and a downregulation of the expression of various other relevant genes [18,89,90]. It could be that excess pesticide exposure exacerbates ROS generation in dragonfly larvae, leading to mitochondrial membrane rupture and reduced transcript levels of mitochondrial PCGs [80,83,91]. This is also consistent with the reported mitochondrial dysfunction when exposed to CPF, with the appearance of fragmented mitochondrial structures [16]. The genes of *A. parthenope* to CPF showed sensitivity, as seen in Figure 1, which is likely due to various factors, including genetic differences, metabolic pathways, and detoxification mechanisms specific to each of the three species analyzed [65,92]. Whereas *A. parthenope* tends to cling to submerged vegetation, other species like *E. elegans* and *G. confluens* burrow or lie flat on the bottom substrate [92]. Several studies have revealed that aquatic insects that inhabit aquatic plants may experience reduced adverse effects from poisons due to the barrier effect of plants [93,94]. Conversely, bottom-dwelling insects are susceptible to pollutants accumulated in sediments and frequently exhibit a certain resistance to poisons [95]. This may explain why *A. parthenope* mitochondrial gene expression differs from that of other species.

## 5. Conclusions

In this study, after 24 h exposure to 0.05 μg/L CPF stress treatment, the transcript levels of several mitochondrial genes were significantly up-regulated in a species-specific pattern. Notably, the *ND4* gene exhibited significant up-regulation of transcript levels in all three dragonfly species under chlorpyrifos exposure, suggesting that the sensitivity of *ND4* to OP pesticides in insects is highly significant compared to other genes. The increased expression of genes associated with these ETC complexes may reflect an adaptive response to enhance respiratory rates and may contribute to resistance against low concentrations of CPF. Furthermore, by examining the effects of higher concentrations of CPF (0.5 µg /L and 5 µg /L) on transcript levels, it was found that, except for *A. parthenope*, that showed a significant decrease in transcript levels and increased larval mortality under 5 µg /L CPF stress, the transcript levels of all other genes remained significantly upregulated in the other species. This may indicate a protective or stress-responsive role in coping with CPF stress. Conversely, the larval mortality of *A. parthenope* and the down-regulation of genes may indicate that mitochondrial function is affected under CPF stress. These findings illuminate the effects of CPF on mitochondrial function in aquatic insects and provide new directions for pesticide detection in aquatic environments, exploring the potential of mitochondrial gene expressions as effective biomarkers for CPF.

## Figures and Tables

**Table 1:** Characteristics of larvae from three dragonfly species in the last stadium.

Species	Width of Head (mm)	Total Length (mm)	Reference
*A. parthenope*	8.43 ± 0.45	45.50 ± 0.25	[67]
*E. elegans*	6.84 ± 0.36	33.7 ± 0.11	[66]
*G. confluens*	7.82 ± 0.39	26.4 ± 0.10	[68]

## Data Availability

Data to support this study are available from the National Center for Biotechnology Information (https://www.ncbi.nlm.nih.gov) (accessed on 20 December 2024). The GenBank numbers are PP577161-PP577163.
